# Anti-fibrotic Potential of WJ-MSC Exosomes in Liver Fibrosis: Mechanistic Insights and Dose-Response Efficacy

**DOI:** 10.5812/ijpr-149480

**Published:** 2024-10-09

**Authors:** Azam Khedri, Mohammadreza Roshanazadeh, Mahdi Hatami, Arash Sanaei, Sahar Saki, Samaneh Salehipour Bavarsad

**Affiliations:** 1 Cellular and Molecular Research Center, Medical Basic Sciences Research Institute, Ahvaz Jundishapur University of Medical Sciences, Ahvaz, Iran

**Keywords:** PDGF-BB, p-AKT, WJ-MSCs, Exosome, Liver Fibrosis

## Abstract

**Background:**

Hepatic fibrosis is a biological response characterized by the accumulation of extracellular matrix (ECM) during the wound healing process. Hepatic stellate cells (HSCs) play a pivotal role in fibrogenesis, transitioning from quiescent to myofibroblast cell types and leading to excessive ECM production. Platelet-derived growth factor (PDGF), a potent mitogen, is produced by activated HSCs, stimulating cell proliferation and migration.

**Objectives:**

This study aims to analyze the impact of Wharton's jelly mesenchymal stem cell (WJ-MSC)-derived exosomes on HSC activation induced by PDGF during liver fibrosis.

**Methods:**

Hepatic stellate cells-T6 cells were treated with PDGF-BB for 24 hours to induce activation, followed by treatment with varying concentrations of WJ-MSC-derived exosomes (0, 25, and 50 µg/mL) for another 24 hours. The effects of exosome treatment on HSC activation were evaluated through flow cytometry, differentiation assays, dynamic light scattering (DLS), transmission electron microscopy (TEM), RT-PCR, and western blot analysis.

**Results:**

Our study yields promising results, highlighting the potential therapeutic effects of WJ-MSC-derived exosomes on liver fibrosis. The dose-dependent decrease in fibrotic markers such as α-SMA, COLA1, and phosphorylated AKT protein in PDGF-BB-treated HSC-T6 cells suggests that WJ-MSC exosomes exert an anti-fibrotic effect by inhibiting HSC activation.

**Conclusions:**

These findings suggest that exosomes derived from WJ-MSCs hold therapeutic promise for liver fibrosis treatment by targeting key pathways involved in HSC activation and fibrogenesis. Further investigation into the underlying mechanisms of this anti-fibrotic effect and the potential clinical applications of WJ-MSC-derived exosomes in liver fibrosis management is warranted.

## 1. Background

Hepatic fibrosis is a biological response characterized by the accumulation of extracellular matrix (ECM) as part of the wound healing process (1, 2). During fibrogenesis, hepatic stellate cells (HSCs) lead to excessive production of ECM and transition from quiescent HSCs to a myofibroblast cell type (3). Activated HSCs are known to produce platelet-derived growth factor (PDGF), which stimulates the proliferation and migration of various cell types. PDGF plays a pivotal role in the activation and proliferation of HSCs during hepatic fibrosis (4, 5).

The PDGF family consists of PDGF-A, B, C, and D, which function as homodimers or heterodimers and contribute to fibrogenesis through their receptors, PDGFR-α and PDGFR-β (6). Platelet-derived growth factor is a crucial pathway in liver fibrosis in both human and animal models. Increased levels of PDGF ligands and receptors in liver fibrosis lead to HSC activation, making the blocking of PDGF signaling an important therapeutic goal (5). The PDGF signaling pathway activates the Phosphatidyl 3-kinase (PI3-kinase)-AKT (Protein kinase B) pathway in HSCs, regulating various cellular processes, including the expression of fibrotic markers like collagen (4, 7). Platelet-derived growth factor binding to the PDGF receptor on the cell surface activates several signaling cascades in HSCs, including the PI3-kinase-AKT pathway (8), which influences gene expression and cellular responses associated with fibrosis. The cross-talk between PDGF signaling and the PI3K/AKT pathway in HSCs contributes to the development of liver fibrosis (4, 9). Expression of the PDGF-BB ligand and its receptor increases in liver fibrosis (6, 10), with PDGF-BB inducing PDGFR-β dimerization (11).

Liver transplantation requires alternative methods due to its inherent limitations. The use of mesenchymal stem cells (MSCs) for tissue regeneration and liver repair in various liver diseases, including fibrosis, has emerged as a promising approach. Mesenchymal stem cells have the capacity to repair and regenerate tissues by differentiating into multiple cell types, secreting paracrine factors, modulating immune responses, and reducing inflammation and fibrosis (2, 9). While MSCs show promising therapeutic potential for liver fibrosis and other liver diseases, there are concerns and risks associated with cell transplantation. Potential risks include tumorigenesis and immune rejection of transplanted MSCs by the recipient's immune system. The paracrine effects of MSCs are mediated by the secretion of various soluble factors and extracellular vesicles (EVs), including exosomes, which have demonstrated therapeutic effects in various diseases, including liver fibrosis (12, 13). Utilizing MSC-derived secretions, such as conditioned media or isolated EVs, as cell-free therapy offers advantages over cell transplantation and has garnered considerable attention. This approach eliminates risks associated with cell transplantation, such as immune rejection and tumorigenesis, while still harnessing the therapeutic properties of MSCs (14).

## 2. Objectives

The primary aim of this experiment is to explore the impact of exosomes derived from Wharton's jelly mesenchymal stem cells (WJ-MSCs) on the activation of the HSC-T6 cell line treated with PDGF-BB, serving as a model to study liver fibrosis in vitro.

## 3. Methods

### 3.1. Reagents

The reagents used in this study included PDGF-BB obtained from Sigma-Aldrich (Germany), RIPA buffer from Sigma-Aldrich (St. Louis, MO, USA), fetal bovine serum (FBS) from Gibco (USA), and penicillin-streptomycin antibiotics from Idea-zist (Iran). Additionally, PE-conjugated mouse anti-human antibodies (CD34, CD45, CD44, and CD105) were sourced from eBioscience, and the EXOCIB isolation kit was procured from CIB Biotech Co (Iran).

### 3.2. Examination of Wharton's Jelly Mesenchymal Stem Cells Differentiation

WJ-MSCs are typically considered optimal for differentiation studies, as they have undergone several rounds of cell division, retaining their stem cell characteristics while remaining responsive to differentiation stimuli. To assess differentiation, WJ-MSCs are seeded into the wells of a 6-well plate, and osteogenic and adipogenic factors are introduced into the culture medium to induce differentiation towards osteogenic (bone) and adipogenic (fat) lineages, respectively. These factors typically consist of specific growth factors, hormones, and supplements that promote stem cell differentiation into the desired cell types. The cells are then incubated in the differentiation-inducing medium for 21 days, allowing sufficient time for WJ-MSCs to differentiate and acquire osteoblast (bone-forming cells) or adipocyte (fat cell) characteristics. After the 21-day differentiation period, the cells are assessed using staining for specific markers associated with osteogenic and adipogenic differentiation.

### 3.3. Determination of Wharton's Jelly Mesenchymal Stem Cells Surface Markers

Flow cytometry, utilizing monoclonal antibodies targeting specific surface markers, is a standard method for characterizing and identifying cell populations, including WJ-MSCs. Wharton's jelly mesenchymal stem cells are trypsinized to detach them from the culture dish, creating a single-cell suspension. After trypsinization, the cells are washed with PBS to remove any remaining trypsin and cellular debris, ensuring a clean and uniform suspension for antibody staining. The cells are then incubated with monoclonal antibodies labeled with phycoerythrin (PE), targeting specific surface markers such as CD34, CD45, CD44, and CD105 (15).

### 3.4. Exosome Isolation

Once the WJ-MSC cells reach the desired density, the FBS concentration in the medium is gradually reduced every 2 days until it reaches 0%. Subsequently, the culture medium is aspirated, and the FBS-free supernatant from the WJ-MSC cells is collected after 72 hours. The conditioned medium is then centrifuged at 3000 rpm for 10 minutes to remove dead cells and debris. Exosomes are isolated from the FBS-free supernatant according to the protocol provided by the EXOCIB kit (CIB Biotech Co) (15).

### 3.5. Exosome Characterization

For transmission electron microscopy (TEM) analysis, the exosome pellet is fixed with a 1% glutaraldehyde solution. The supernatant is discarded, and a few drops of the solution are placed on grids, followed by staining with uranyl acetate dye. Photographs are captured using an electron microscope (Zeiss - EM10C - 100 KV) at an acceleration voltage of 100 KV. To determine exosome diameter using the Dynamic Light Scattering (DLS) technique with the SCATTER SCOPE device, the size of particles in 100 microliters of phosphate buffer is analyzed through radiation and light scattering.

### 3.6. Treatment of Hepatic Stellate Cells-T6 Cell Culture

Hepatic stellate cells-T6 cells are cultured at a density of 1×10^6^ cells/well. They are maintained in complete DMEM medium (10% FBS and 100 U/L penicillin-streptomycin) and incubated at 37°C with 5% CO_2_ for 24 hours (16). Subsequently, HSC-T6 cells are activated with 10 ng/mL of PDGF-BB for 24 hours. PDGF-BB is dissolved in DMEM containing 2% FBS (8, 17). The HSC-T6 cells are divided into four groups: Control group: HSC-T6 cell line without stimulation; PDGF-BB group: HSC-T6 cells stimulated with 10 ng/mL of PDGF-BB; exosome (25 µg/mL) group: HSC-T6 cells stimulated with PDGF-BB and treated with exosomes at a concentration of 25 µg/mL; exosome (50 µg/mL) group: HSC-T6 cells stimulated with PDGF-BB and treated with exosomes at a concentration of 50 µg/mL.

### 3.7. Real-time PCR

Following the 24-hour treatment period, the cells are digested, and total RNA is extracted using a commercial kit (Yekta Tajhiz Azma, IRAN). This kit typically includes reagents for cell lysis, RNA binding, washing, and elution to isolate high-quality RNA from the cells. The extracted total RNA is then reverse transcribed into complementary DNA (cDNA) using reverse transcriptase enzyme and primers provided in the kit, following the manufacturer's instructions. The synthesized cDNA serves as a template for RT-PCR amplification using SYBR^®^ Green Master Mix, enabling real-time monitoring of PCR amplification. During amplification, specific primer sequences designed to target the genes of interest are used, with GAPDH serving as an internal reference gene for normalization. The primer sequences were designed by Sinaclon Company (Tehran, Iran) (Table 1). 

**Table 1.** Primer Sequence for RT-PCR

Gene	Primers Sequence	Size of PCR Product (bp)
**COLA1 **	F. 5′-TGAAGGGACACAGAGGTTCA-3′	188
**COLA1 **	R. 5′-ACCATCATTTCCACGAGCA-3′	
**α-SMA**	F. 5′-CAAGTCCTCCAGCGTTCTGA-3′	196
**α-SMA **	R. 5′-GCTTCACAGGATTCCCGTCTT-3′	
**E-Cadherin**	F. 5′- TGGTTCAAGCTGCTGACCTT-3′	178
**E-Cadherin**	R. 5′- CTGACCCTTGTACGTGGTGG-3′	
**N-Cadherin**	F. 5′-AGGCTTCTGGTGAAATCGCA-3′	198
**N-Cadherin**	R. 5′-AAATCTGCAGGCTCACTGCT-3	
**GAPDH**	F. 5′-TCGGAGTCAACGGATTTGGT-3′	181
**GAPDH**	R. 5′-TTCCCGTTCTCAGCCTTTGAC-3′	

Abbreviations: RT-PCR, reverse transcription-polymerase chain reaction; COLA1; collagen type I; α-SMA; alpha-smooth muscle actin; GAPDH, glyceraldehyde 3-phosphate dehydrogenase; F, forward; R, reverse.

### 3.8. Western Blot Technique

Hepatic stellate cells are lysed with RIPA buffer on ice to extract proteins while minimizing degradation. The extracted proteins are then separated by molecular weight using SDS-PAGE. Afterward, the proteins are transferred onto a PVDF membrane, which is then incubated with primary antibodies specific to the target proteins of interest. Following the removal of unbound primary antibodies, the membrane is incubated with enzyme-conjugated secondary antibodies for signal amplification. The protein bands bound by the antibodies are visualized using a ChemiDoc imaging system, which captures images of the chemiluminescent signals emitted by the ECL (enhanced chemiluminescence) reagent reacting with the enzyme-labeled secondary antibodies.

### 3.9. Statistical Analysis

Data are presented as means ± SEM and analyzed using ANOVA followed by the Tukey test with GraphPad Prism 9.0.1 software. Statistical significance is considered with a P-value less than 0.05.

## 4. Results

### 4.1. Features of Stem Cells

**Figure 1.**
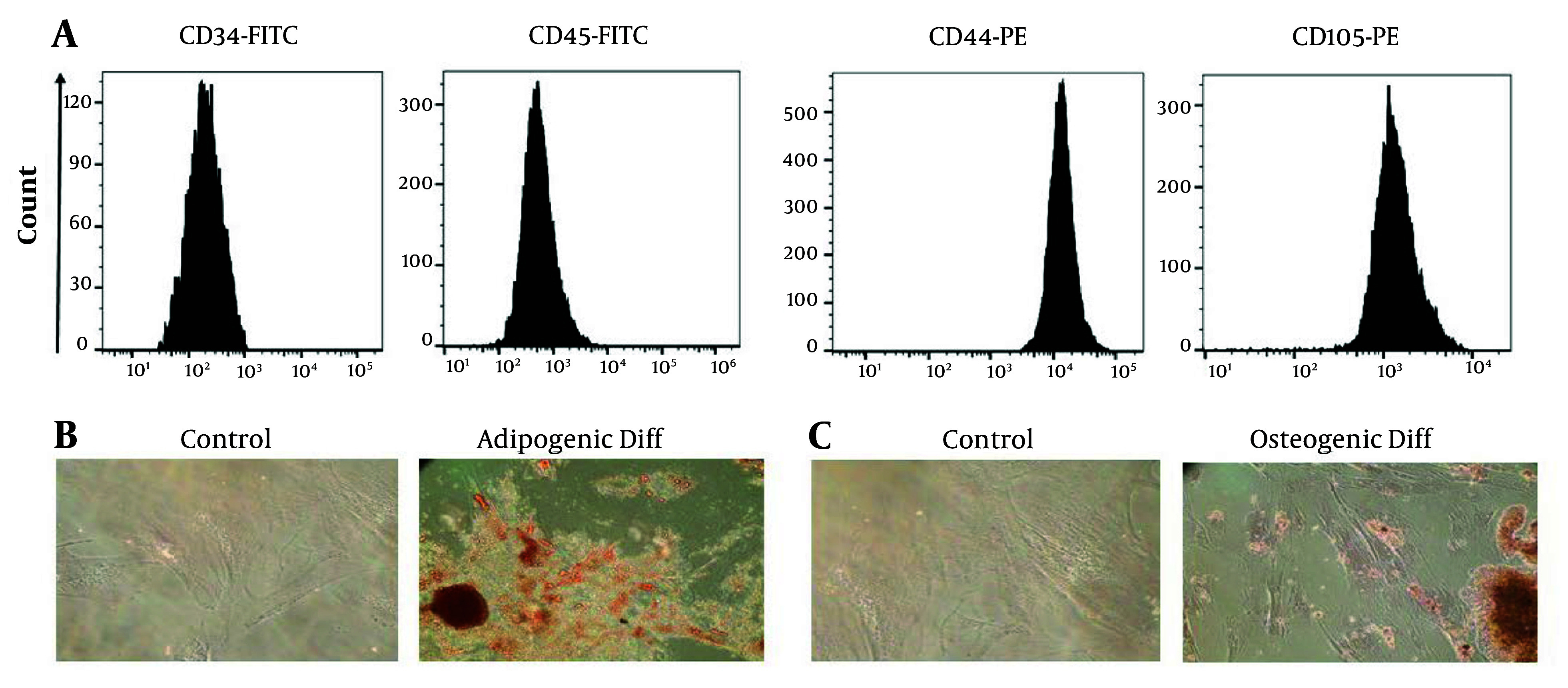
Examining the expression of surface markers of Wharton's jelly mesenchymal stem cells (WJ-MSCs) using flow cytometry and differentiation of WJ-MSCs into adipogenic and osteogenic tissue. A, the WJ-MSCs were positive for CD44 and CD105 markers and negative for CD34 and CD45 markers; B, to check the differentiation into adipocytes, the cells were exposed to Oil Red O dye, and by observing lipid droplets, the differentiation into adipocytes was confirmed; C, the observation of calcium deposits after Alizarin Red S staining indicates successful differentiation of the WJ-MSCs into osteoblasts, highlighting their potential for osteogenic differentiation.

Under light microscopy, the cells appeared spindle-shaped and adhesive. Flow cytometry analysis confirmed the cells as MSCs, as they were positive for CD44 and CD105 and negative for CD34 and CD45 surface markers (Figure 1A). Further characterization of the cells involved differentiation assays. Adipogenic differentiation was confirmed by exposing the cells to Oil Red O dye, which showed the presence of lipid droplets characteristic of adipocyte differentiation (Figure 1B). Osteogenic differentiation was confirmed by exposing the cells to Alizarin Red S dye, which revealed calcium deposits indicative of osteoblast differentiation (Figure 1C). Passage 3 cells were used for exosome isolation. 

### 4.2. Exosome Characterization

**Figure 2.**
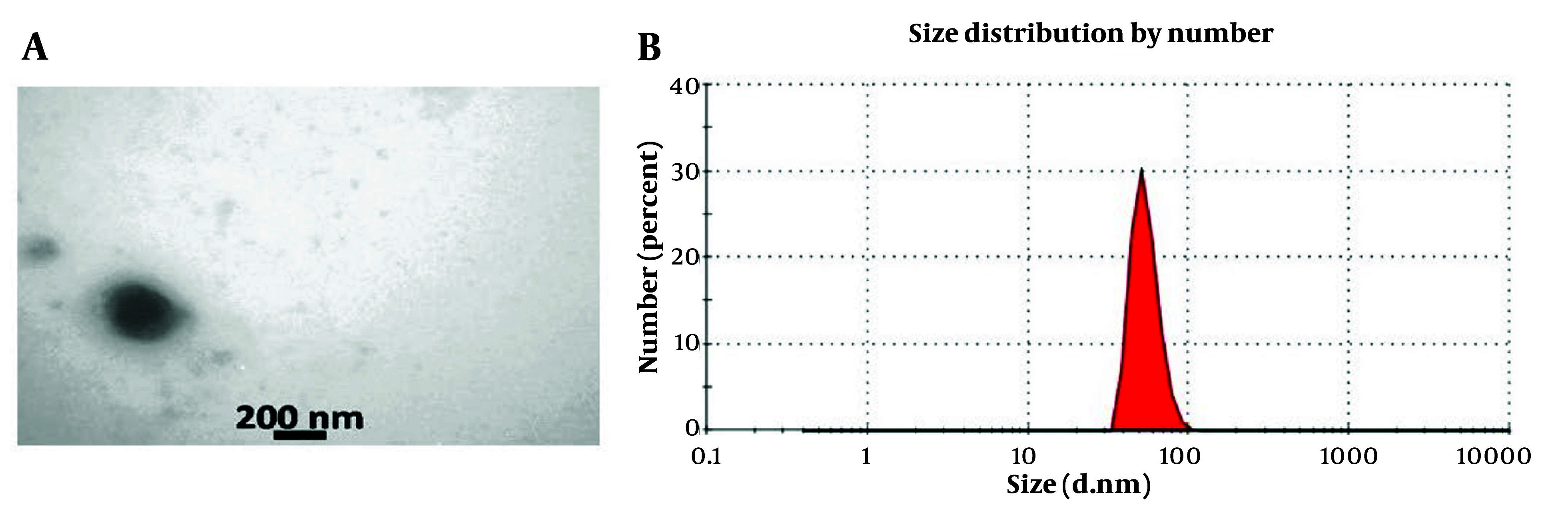
A, microscopic image of exosome with uranyl acetate staining transmission electron microscopy (TEM) technique. In this image, the double-layered membrane of exosomes is clear; B, measuring the size of exosomes by DLS

The exosome structure was examined using TEM, which revealed intact membranes and spherical structures characteristic of exosomes. Transmission electron microscopy analysis showed that the exosomes exhibited a spherical morphology with a lipid bilayer (A, microscopic image of exosome with uranyl acetate staining TEM technique. In this image, the double-layered membrane of exosomes is clear; B, measuring the size of exosomes by DLSure Figure 2A). The diameter of the exosome particles in the suspension was determined using DLS with the Nanosizer device, revealing an average diameter of 73 nm (Figure 2B). 

### 4.3. Activating of Hepatic Stellate Cells-T6 Cells by PDGF-BB

**Figure 3.**
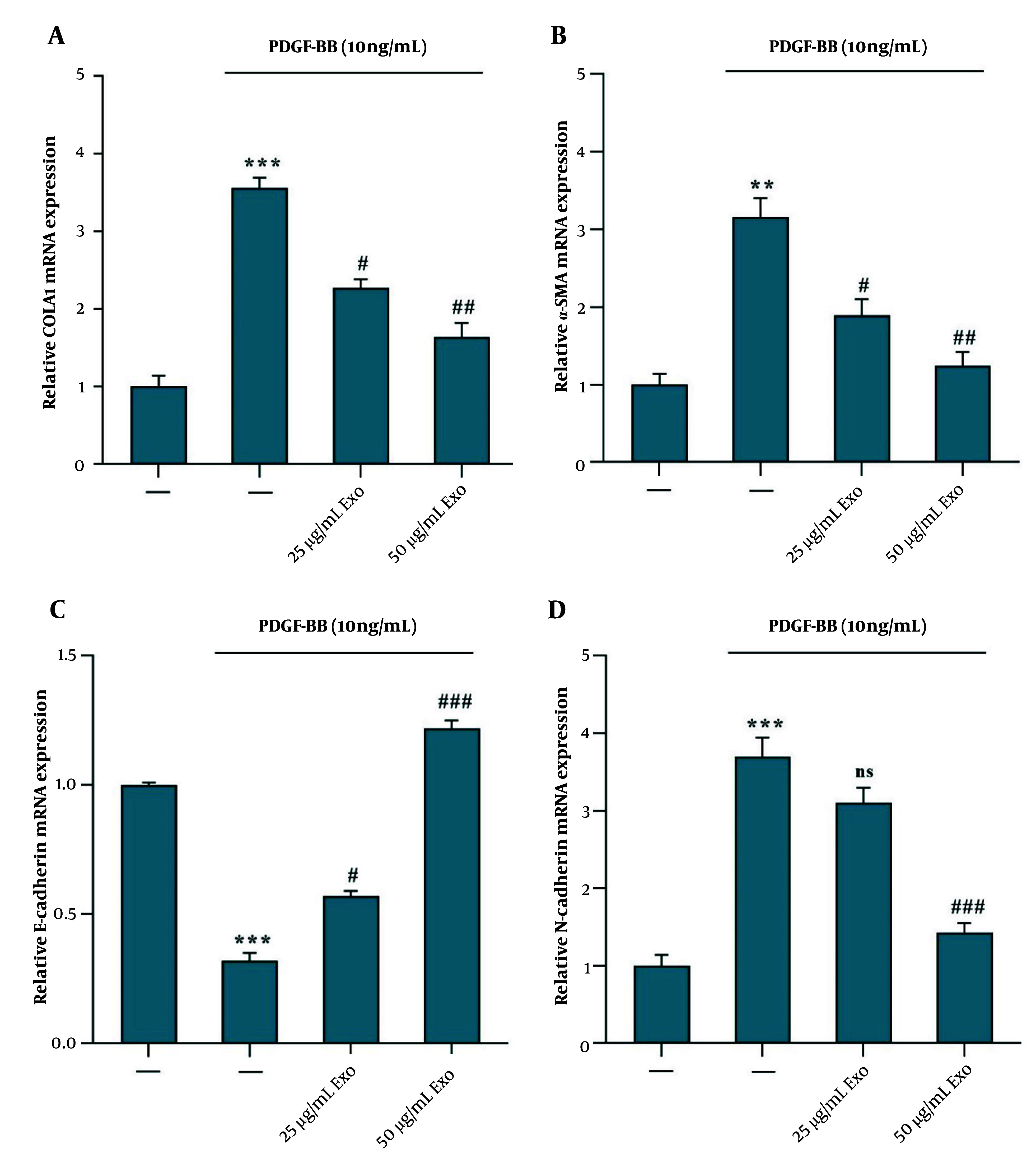
Exosomes derived from WJ-MSC reduce α‑SMA, COLA1, and N-Cadherin mRNA expression and increase E-Cadherin mRNA expression in HSC‑T6 stimulated by 10 ng/mL PDGF-BB. The stimulated HSC‑T6 cells were treated with two concentrations of WJ-MSC-derived exosomes (25 and 50 μg/mL) for 24 hours. The mRNA expression levels were detected via RT-PCR for the following: A, COLA1, effect of exosomes vs. PDGF-BB; B α‑SMA, effect of exosomes vs. PDGF-BB; C, E-Cadherin, effect of exosomes vs. PDGF-BB; and D N-Cadherin, effect of exosomes vs. PDGF-BB. Results are presented as mean ± SEM. ** P < 0.01, *** P < 0.001, # P < 0.05, ## P < 0.01, ### P < 0.001 vs. control group. Abbreviations: WJ-MSC, Wharton's jelly mesenchymal stem cells; HSC, hepatic stellate cell; PDGF, platelet-derived growth factor; Exo, exosomes of WJ-MSCs; α‑SMA, α‑smooth muscle actin; COLA1, collagen type I α 1.

The results indicated that PDGF-BB treatment led to the activation of HSC-T6 cells. The upregulation of COLA1, α-SMA, and N-Cadherin gene expression suggests increased ECM production and myofibroblast activation, as indicated by the elevated levels of α-SMA and N-Cadherin (Figure 3A, B, and D). Treatment of activated HSC-T6 cells with exosomes derived from WJ-MSCs at concentrations of 25 and 50 μg/mL for 24 hours resulted in a downregulation of mRNA expression of COLA1 (Figure 3A), α-SMA (Figure 3B), and N-Cadherin (Figure 3D) genes. Additionally, there was an observed increase in E-Cadherin mRNA expression, indicating a reversal effect on the mesenchymal markers (Figure 3C). This suggests that WJ-MSC-derived exosomes may exert inhibitory effects on the fibrogenic and myofibroblastic phenotypes induced by PDGF-BB activation in HSC-T6 cells.

### 4.4. Phosphorylation of AKT Protein in PDGF-BB Treated Hepatic Stellate Cells-T6 with Exosomes of Wharton's Jelly Mesenchymal Stem Cell 

**Figure 4.**
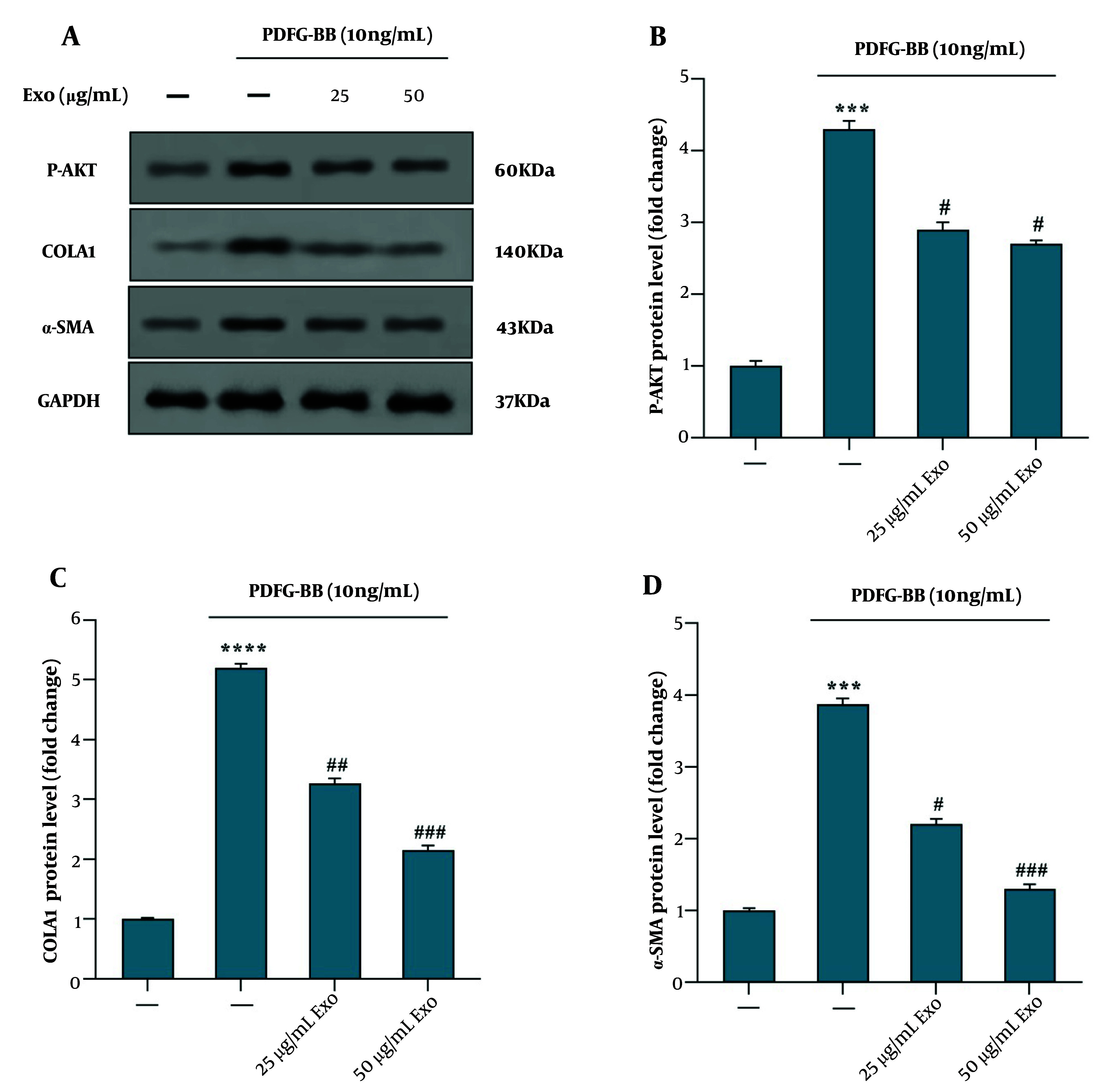
Effect of exosomes derived from WJ-MSC on the phosphorylation of the AKT signaling pathway in HSC‑T6 stimulated by 10 ng/mL PDGF‑BB for 24 hours. A, the protein levels of p‑AKT, COLA1, α‑SMA, and GAPDH were detected using western blotting; B, the relative p‑AKT protein level is presented as a fold change, showing the effect of exosomes vs. PDGF‑BB; C, the protein levels of COLA1, showing the effect of exosomes vs. PDGF‑BB; D, the protein levels of α‑SMA, showing the effect of exosomes vs. PDGF‑BB. Results are presented as mean ± SEM. *** P < 0.001, **** P < 0.0001, # P < 0.05, ## P < 0.01, ### P < 0.001 vs. control group. Abbreviations: WJ-MSC, Wharton's jelly mesenchymal stem cells; HSC, hepatic stellate cell; PDGF, platelet-derived growth factor; Exo, exosomes of WJ-MSCs; α‑SMA, α‑smooth muscle actin; COLA1, collagen type I α 1; GAPDH, glyceraldehyde 3-phosphate dehydrogenase.

The western blot analysis data revealed that pretreatment of HSC-T6 cells with exosomes derived from WJ-MSCs at concentrations of 25 and 50 μg/mL for 4 hours, followed by PDGF-BB stimulation, resulted in decreased phosphorylation of Akt (P-Akt) and reduced levels of COLA1 and α-SMA proteins, compared to cells treated with PDGF-BB alone (Figure 4A). Exosome treatment at both concentrations (25 and 50 μg/mL) significantly downregulated AKT phosphorylation (Figure 4B). Furthermore, the reduction in COLA1 (Figure 4C) and α-SMA (Figure 4D) protein levels was more pronounced at the higher concentration of exosomes (50 μg/mL) compared to the lower concentration (25 μg/mL), suggesting a dose-dependent inhibitory effect on fibrotic marker expression.

## 5. Discussion

Liver fibrosis is a significant global health concern and represents a common pathway for chronic liver diseases that can lead to cirrhosis, liver failure, and hepatocellular carcinoma. Hepatic stellate cells play a central role in the pathogenesis of liver fibrosis, undergoing activation from a quiescent state to a myofibroblastic phenotype characterized by excessive ECM production (18). This transition is primarily driven by pro-fibrogenic cytokines like platelet-derived growth factor-BB (PDGF-BB) (19).The PDGF-BB is recognized for its mitogenic and proliferative effects on HSCs, promoting their activation and fibrogenic responses (17, 19). Recent advances in regenerative medicine have highlighted the potential of MSCs and their secreted exosomes in modulating fibrogenic pathways. Exosomes, as nano-sized EVs, carry a cargo of bioactive molecules capable of influencing the behavior of recipient cells (18, 19).

This study aimed to investigate the characteristics of MSCs, the effect of PDGF-BB on the activation of HSC-T6, and the potential therapeutic effects of exosomes derived from WJ-MSCs on PDGF-BB-activated HSC-T6 cells. Our findings describe the morphological and phenotypic properties of MSCs, the fibrogenic activation of HSC-T6 cells under PDGF-BB stimulation, and the inhibitory effects of WJ-MSC-derived exosomes on fibrogenic markers and signaling pathways.

Although various compounds, such as Inonotsuoxide B (20) and Plantamajoside (17), have been used to inhibit HSC activation via different pathways, such as PI3K/AKT, no studies have examined the inhibitory effects of WJ-MSC-derived exosomes on HSC activation. By stimulating HSC-T6 cells with PDGF-BB and treating them with WJ-MSC-derived exosomes at different concentrations (25 and 50 µg/mL), we aim to investigate the anti-fibrotic effects of these exosomes and elucidate their potential mechanisms, which may include modulation of inflammatory responses, inhibition of HSC activation pathways (such as PDGF-BB signaling), promotion of HSC apoptosis or senescence, and regulation of ECM remodeling processes.

Understanding the intrinsic characteristics of MSCs, particularly their capacity to modulate the behavior of recipient cells, is crucial for maximizing their therapeutic efficacy. The differentiation potential of these cells was validated through osteogenic and adipogenic assays, demonstrating their ability to differentiate into osteoblasts and adipocytes, respectively. These findings align with previous studies that have characterized MSCs based on their morphology, surface markers, and differentiation potential (21, 22). Transmission electron microscopy revealed that WJ-MSC-derived exosomes exhibited intact membranes and spherical structures, characteristic of exosomes. Dynamic light scattering analysis further confirmed the average diameter of the exosomes to be 73 nm, which is within the typical size range for exosomes. Similar studies have reported comparable morphological features and size distributions for MSC-derived exosomes, reinforcing the reliability of our characterization methods (23, 24).

Our study indicates that PDGF-BB treatment resulted in the activation of HSC-T6 cells, as evidenced by the upregulation of COLA1, α-SMA, and N-Cadherin gene expression. These markers are indicative of ECM production and myofibroblast activation, hallmark features of fibrogenesis (3, 17, 25, 26). Additionally, the observed decrease in E-Cadherin expression suggests a shift towards a mesenchymal phenotype, a process known as epithelial-to-mesenchymal transition (EMT), which is pivotal in liver fibrosis (27). Furthermore, our investigation reveals that PDGF-BB-stimulated HSC-T6 cells exhibit P-Akt, a key signaling molecule involved in HSC proliferation and collagen production. This observation is consistent with existing literature linking Akt activation to elevated collagen mRNA and protein levels in HSCs, highlighting its role in promoting fibrotic processes (8).

Treatment with WJ-MSC-derived exosomes at concentrations of 25 and 50 µg/mL resulted in a significant downregulation of COLA1, α-SMA, and N-Cadherin gene expression in PDGF-BB-activated HSC-T6 cells. This suggests that the exosomes possess anti-fibrotic and anti-myofibroblastic properties, capable of reversing the PDGF-BB-induced fibrogenic phenotype. Notably, the effect was dose-dependent, with the 50 µg/mL concentration producing more pronounced reductions in fibrotic marker expression. These findings are in line with other studies demonstrating the therapeutic potential of MSC-derived exosomes in modulating fibrosis and reducing ECM production (28).

Western blot analysis revealed that pre-treatment with WJ-MSC-derived exosomes resulted in decreased P-Akt in PDGF-BB-stimulated HSC-T6 cells, along with reduced levels of COLA1 and α-SMA proteins. This suggests that the exosomes exert their anti-fibrotic effects, at least in part, by inhibiting the AKT signaling pathway, which plays a critical role in cell survival, proliferation, and fibrogenesis (29). The reduction in AKT phosphorylation upon treatment with WJ-MSC exosomes indicates potential regulation of the PI3K/AKT signaling pathway, which is central to cell generation and fibrogenesis. Inhibition of AKT phosphorylation may lead to decreased activation of downstream signaling cascades associated with fibrosis development, further supporting the anti-fibrotic effects of WJ-MSC exosomes. These results suggest that WJ-MSC exosomes exert multi-faceted anti-fibrotic effects on HSC-T6 cells by targeting key molecular pathways involved in fibrogenesis, including HSC activation, ECM deposition, EMT, and signaling pathways.

Several factors are involved in HSC activation, functioning through different pathways. Various inflammatory factors, such as IL-1β, TNF-α, and IFN-γ, are reported to activate HSCs (30). Zhang et al. reported that MSC exosomes suppressed inflammation and other effects induced by IL-1β actions (31). Moreover, the studies by Eshghi et al. and Ma et al. demonstrated the inhibitory effects of MSC exosomes on inflammatory factors, including IL-6, IL-1β, and TNF-α (32, 33). TGF-β, a major growth factor, plays a critical role in various cellular functions, such as proliferation and cell growth. It functions as an activator of HSCs, primarily through Smad signaling (34). The works of Hu et al. and Didamoony et al. suggest that MSC exosome treatment results in the suppression of TGF-β/Smad signaling. Didamoony reported that miR-200a plays an important role as a mediator for the function of MSC exosomes in regulating TGF-β signaling (35, 36). Additionally, Zhang et al.'s research suggests that miR-21-5p and miR-125b-5p act as mediators for MSC exosomes, inhibiting TGF-β type II and type I receptors, respectively (37).

Integrins are known as major ECM receptors, composed of heterodimers of α and β subunits. The role of integrins in HSC activation is mediated through various extracellular ligands, including TGF-β, which transmit signals from the cell membrane to the cytoplasm, activating signaling pathways such as MAPK and PI3K/Akt (38, 39). Li et al. reported that MSC-exosomal miR-3940-5p could suppress the process of epithelial-mesenchymal transition (EMT) in CRC cells by targeting integrin A6 (40). There is a wide variety of activating factors for HSCs that lead to liver fibrosis, and each of these factors could potentially be targeted by MSC exosomes, resulting in the regression of liver fibrosis. However, further studies are required to clarify the specific functions of WJ-MSC exosomes in liver fibrosis.

The inhibition of gene and protein expression by WJ-MSC-derived exosomes at the 50 µg/mL concentration further underscores the potential utility of optimizing exosome concentration for therapeutic purposes. Our observation of the dose-dependent effects of WJ-MSC-derived exosomes in ameliorating hepatic fibrosis is noteworthy and aligns with the concept of optimal therapeutic dosing in regenerative medicine. The finding that the higher dose of exosomes (50 µg/mL) was more effective in mitigating fibrosis compared to the lower dose (25 µg/mL) suggests a potential dose-response relationship, where a higher concentration of exosomes may yield more pronounced anti-fibrotic effects.

The enhanced efficacy of the higher exosome dose could be attributed to several factors, including increased delivery of bioactive molecules and signaling factors present in the exosomes, which may exert stronger regulatory effects on HSC activation and fibrotic marker expression. Additionally, at a higher concentration, exosomes may interact with a greater number of target cells and modulate signaling pathways more robustly, thereby augmenting their overall therapeutic impact on hepatic fibrosis. These findings emphasize the importance of optimizing exosome dosing regimens in the context of liver fibrosis treatment and suggest that higher concentrations of WJ-MSC-derived exosomes may offer enhanced benefits in combating fibrotic processes. Further elucidation of the mechanisms underlying the dose-dependent effects of exosomes on fibrosis attenuation will provide valuable insights for refining therapeutic strategies aimed at harnessing the therapeutic potential of WJ-MSC-derived exosomes in liver fibrosis management.

### 5.1. Conclusions

Our study findings suggest that exosomes derived from WJ-MSCs can inhibit PDGF-BB-stimulated HSC activation. This inhibition appears to be dose-dependent and is associated with the suppression of the PI3K/AKT signaling pathway, as indicated by reduced p-AKT levels. Furthermore, the decreased expression of fibrotic markers, including COL1A1, α-SMA, and N-cadherin, along with the increase in E-cadherin, suggests that WJ-MSC exosomes effectively impede HSC activation and survival.

## Data Availability

The dataset presented in the study is available on request from the corresponding author during submission or after publication.
